# Mixed Two-Dimensional Organic-Inorganic Halide Perovskites for Highly Efficient and Stable Photovoltaic Application

**DOI:** 10.3390/molecules24112144

**Published:** 2019-06-06

**Authors:** Jia-Yi Dong, Zi-Qian Ma, Ye Yang, Shuang-Peng Wang, Hui Pan

**Affiliations:** 1 Joint Key Laboratory of the Ministry of Education, Institute of Applied Physics and Materials Engineering, University of Macau, Macao SAR 999078, China; yb67446@connect.um.edu.mo (J.-Y.D.); yeyang@um.edu.mo (Y.Y.); 2 School of Mechanical Engineering, Zhuhai College of Jilin University, Zhuhai 519000, China; Yb47433@connect.um.edu.mo; 3 Department of Physics and Chemistry, Faculty of Science and Technology, University of Macau, Macao SAR 999078, China

**Keywords:** mixed 2D HOIPs, solar-energy harvesting, electronic properties, effective mass, stability, first-principles calculation

## Abstract

Solar cells made of hybrid organic-inorganic perovskite (HOIP) materials have attracted ever-increasing attention due to their high efficiency and easy fabrication. However, issues regarding their poor stability remain a challenge for practical applications. Engineering the composition and structure of HOIP can effectively enhance the thermal stability and improve the power conversion efficiency (PCE). In this work, mixed two-dimensional (2D) HOIPs are systematically investigated for solar-power harvesting using first-principles calculations. We find that their electronic properties depend strongly on the mixed atoms (Cs, Rb, Ge and Pb) and the formation energy is related to the HOIP’s composition, where the atoms are more easily mixed in SnI-2D-HOIPs due to low formation energy at the same composition ratio. We further show that optimal solar energy harvesting can be achieved on the solar cells composed of mixed SnI-2D-HOIPs because of reduced bandgaps, enhanced mobility and improved stability. Importantly, we find that the mixed atoms (Cs, Rb, Ge and Pb) with the appropriate composition ratios can effectively enhance the solar-to-power efficiency and show greatly improved resistance to moisture. The findings demonstrate that mixed 2D-HOIPs can replace the bulk HOIPs or pure 2D-HOIPs for applications into solar cells with high efficiency and stability.

## 1. Introduction

The discovery of hybrid organic-inorganic perovskites (HOIPs) with three-dimensional (3D) or two-dimensional (2D) crystal structures have shown great potential on solar-energy harvesting/storage technologies (e.g., solar cell, light-emitting diodes (LED), laser, photodetector and energy storage devices) [1,2,3,4,5,6,7,8,9,10,11,12,13,14]. The superior performance of HOIPs in solar-energy harvesting is mainly attributed to their unique and excellent physicochemical properties, such as narrow bandgap, high mobility, efficient separation of photogenerated electron-hole pairs, and low-cost technologies for fabrication [15,16,17,18,19,20]. The power conversion efficiency (PCE) of 3D-HOIPs materials (ABX_3_, where A is CH_3_NH_3_^+^ (MA^+^) or CH(NH_2_)^+^ (FA^+^), B is Pb^2+^ or Sn^2+^ and X is Cl^−^, Br^−^ or I^−^ has dramatically risen from 3.8% in 2009 to 24.2% in 2019 [21,22]. However, the stability remains a challenging issue for commercialization [23,24,25,26,27]. To effectively improve the stability of 3D-HOIPs, composition optimization became a promising approach by modifying their electronic structures [5,28,29,30,31,32]. Recent studies have shown that the composition ratios of mixed lead halide perovskites could markedly enhance thermal stability and improve PCE. For example, Duong et al., found that the multi-cation (MA/FA/Cs/Rb) mixture HOIP system [33] showed a steady state performance of up to 17.4% with negligible hysteresis. Meanwhile, the light stability was also improved significantly upon the addition of Cs and Rb, and the cell retained up to 95% efficiency after 12 h of operation. Similarly, the Sn-based MA_0.5_FA_0.5_Pb_0.75_Sn_0.25_I_3_ perovskite solar cells with a PCE of 14.19% kept 94% efficiency after 30 days in an inert atmosphere and 80% efficiency after 12 days exposed to ambient atmosphere (30–40% RH) [34]. Xu et al. reported that a high-quality MA_0.5_FA_0.5_Pb_0.5_Sn_0.5_I_3_ film effectively improved the stability of HOIP materials against oxygen and significantly reduced leakage current [35]. Syzgantseva et al. demonstrated that Cs and Rb cations were more efficient in the stabilization of the HOIPs than MA cations at ambient temperature [36]. In addition, Zhang et al. found that Cs cations also played a key role on the emission property of the HOIP materials [37]. They found that the optimized composition of FA_0.8_Cs_0.2_PbBr exhibited excellent performance with a high reported luminance of 55,005 cd m^−2^ and a current efficiency of 10.09 cd A^−1^. Besides experimental progress, theoretical studies were also reported. Mosconi et al. investigated a series of mixed Pb–Sn perovskites (MASn*_x_*Pb_(1−*x*)_I_3_) by changing x and found the 50:50 compound showed red-shift and increased absorption spectra along with almost perfectly balanced electron and hole transport properties [38]. Ju et al., found that RbSn_0.5_Ge_0.5_I_3_ exhibited small carrier effective masses and low exciton-binding energies and comparable absorption spectrum of sunlight to the prototype MAPbI_3_ [39].

Despite noticeable and extremely fast progress in the improved stability, almost all of the research on compositional engineering were focused on the 3D-HOIP materials, while for the 2D-HOIPs that show controllable bandgap, multi-functional properties, and high stability [40], more study is still required. However, the low PCE is their bottleneck. Recently, a PCE of 16.6% had been reported on a solar cell with 2D-HOIPs [41]. It was found that 2D-HOIPs with tunable bandgaps can be achieved by replacing organic ions, metal atoms, or halide atoms [42]. As a result, composition optimization may be a promising approach to improve the PCE and stability of 2D HOIPs.

Here, we systemically investigate the effects of substitutional cations (Cs^+^ and Rb^+^) and metal ions (Ge^2+^ and Pb^2+^) with different composition ratios on the structural and electronic properties, and the stability of four 2D-HOIPs systems (SnI-2D-HIOPs, PbI-2D-HIOPs, SnBr-2D-HIOPs, and PbBr-2D-HIOPs) based on first-principles calculations. We find that SnI-2D-HOIPs is easier to mix these atoms than other 2D-HIOPs systems due to low formation energy at the same composition ratio. We show that the mixed atoms (Cs, Rb, Ge and Pb) with the appropriate composition ratios can effectively enhance the performance of 2D HOIPs and their resistance on moisture. We demonstrate that the best performance in solar-to-power conversion can be realized on the solar cells with mixed SnI-2D-HOIPs as light absorber because of the narrowest bandgap, highest mobility and highest stability in all of the considered systems.

## 2. Computational Detail

We carried out an ab initio study on the physical properties of 2D-HOIPS, focusing on (RNH_3_)_2_(MA)_n−1_B_n_X_3n+1_ (R: long-chain alkyl or aromatic group; *n*: the number of metal-halide sheets), and the effects of phase, thickness and surface molecule based on density-functional theory (DFT) [43,44,45,46] and Perdew-Burke-Eznerhof generalized gradient approximation (PBE-GGA) [47,48] to accurately calculate the total energy. The Vienna ab initio simulation package (VASP) with the projector augmented wave (PAW) scheme was used [46,49,50]. The Monkhorst-Pack method was used to generate k-point meshes in the first Brillouin zone [51,52]. A 3 × 3 × 1 grid was used consistently. A nonlocal density functional, vdW-DF [53], was employed to describe a weak interaction between the organic molecule and inorganic matrix. The cut-off energy is 500 eV.

## 3. Results and Discussion

### 3.1. Structural Optimization and Formation Energy

**Figure 1:**
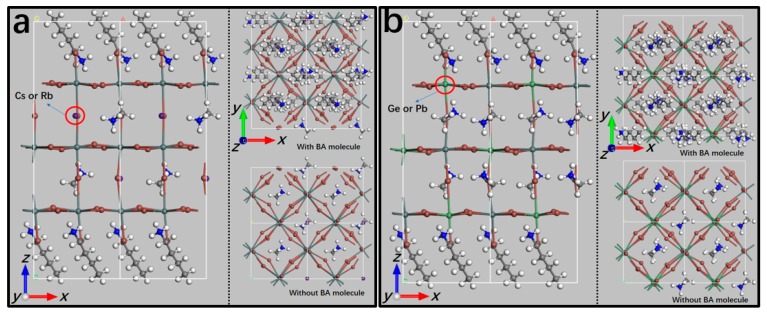
(**a**) Optimized structures of BA_2_MA_2_B_3_X_10_ (B = Pb^2+^ or Sn^2+^; X = Br^−^ or I^−^) with the mixed Cs/Rb atoms. (**b**) Relaxed structures of BA_2_MA_2_Sn_3_I_10_ with the mixed Ge/Pb atoms. Sn/Pb = silver gray; Cs/Rb = purple; Ge/Pb = green; I/Br = bronze; N = blue; C = gray; H = white.

The 2D-HOIPs are directly obtained from the related bulk structures by cutting layers in the <100> direction [54]. The 2D-HOIPs with different phases (tetragonal and orthorhombic) can be obtained due to the orientation of C–N bonds with the MA cations. Our previous study demonstrated that the 2D tetragonal phase with the parallel (TETP) structure of the C-N bonds shows the highest efficiency for solar-to-power conversion [40]. Therefore, in this study, we focus on the TETP structures with three inorganic sheets (*n* = 3) of 2D-HOIPs, including SnI-2D-HOIPs, PbI-2D-HOIPs, SnBr-2D-HOIPs and PbBr-2D-HOIPs. CH_3_(CH_2_)_3_NH_3_^+^ (BA) is employed as functional organic cations to protect their surfaces. The system has the formula of BA_2_MA_2_B_3_X_10_ (Figure 1). In order to explore the composition engineering on 2D-HOIPs, we replace the MA molecule by Cs and Rb atom with four ratios (25%, 50%, 75% and 100%) in these four systems, respectively. Recent report also demonstrated that the mixed 3D-HOIPs with Cs, Rb, Ge and Pb atoms were stable due to their favorable Goldschmidt tolerance factors [38,39]. So, we also investigate the effect of the mixed Ge and Pb atom with five composition ratios (16.67%, 33.33%, 50%, 66.67% and 83.33%) to replace Sn on the structural property of the Sn-based 2D HOIPs system. Our optimized geometries (Supporting data, Tables S1–S4) show that the variations in the lattice constants of these four mixed 2D HOIP systems with the different composition ratios of Cs and Rb atoms all directions range from 0–2.28%. The lattice constants of Sn-based 2D HOIPs with mixed Ge and Pb atoms change within 0–1.99%. As the concentrations of the mixed atoms (Cs, Rb, Ge and Pb) atom increase, the optimized lattice constants of almost all the mixed 2D HOIPs trend to decrease with a few exceptions, such as Pb-based 2D HOIPs mixed with Rb atoms (Tables S1–S4).

**Figure 2:**
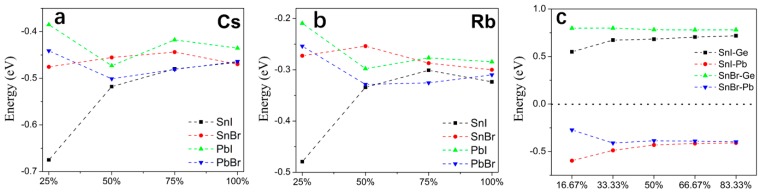
Formation energies of BA_2_MA_2_B_3_X_10_ (B = Pb^2+^ or Sn^2+^; X = Br^−^ or I^−^) mixed with (**a**) Cs atom and (**b**) Rb atom. (**c**) Formation energy of BA_2_MA_2_Sn_3_I_10_ mixed with the Ge or Pb atom.

The formation energies of these four mixed 2D HOIPs systems were investigated to study their formation possibility. The following equation is used to calculate the formation energy:

(1) $$E_{f}=(E_{tot}\left(mixture\right)-E_{tot}\left(pure\right)-n\mu_{X}+n\mu_{Y})/n)$$

where $E_{tot}\left(mixture\right)$ and $E_{tot}\left(pure\right)$ are the total energies of the mixed 2D HOIPs and pure 2D HOIPs, respectively. $\mu_{X}$ is the energy of Cs, Rb, Ge, or Pb atom. $\mu_{Y}$ is the energy of MA molecule or Sn atom. n is the number of substitutional atoms. We find that the formation energies of these four 2D HOIPs systems with the mixed Cs, Rb and Pb atoms are negative, indicating that the binding is stronger, and the incorporation process is exothermic (Figure 2a,b). While the formation energy by mixing Ge atom is positive, indicating that the incorporation is endothermic (Figure 2c). Meanwhile, as the concentration of mixed atoms increase, the formation energies of 2D HOIPs have a tendency to converge. The calculated results clearly show that Cs, Rb, and Pb atoms can easily replace the corresponding MA molecule or Sn atoms in 2D HOIPs under moderate conditions due to the negative substitutional energies, while Ge is not easy to make it because of its large positive formation energy.

### 3.2. Electronic Structures of Mixed 2D-HOIPs

**Figure 3:**
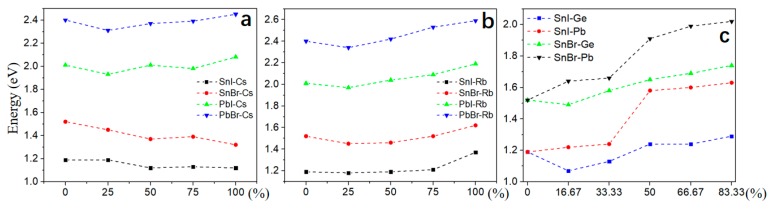
Bandgaps of BA_2_MA_2_B_3_X_10_ (B = Pb^2+^ or Sn^2+^; X = Br^−^ or I^−^) mixed with (**a**) Cs atom and (**b**) Rb atom. (**c**) Bandgaps of BA_2_MA_2_Sn_3_I_10_ mixed with Ge or Pb atom.

Similar to the pure 2D-HOIPs, the mixed 2D-HOIPs are also semiconducting with direct bandgaps, where both the conduction band bottom (CBB) and valence band top (VBT) are at Γ point (Figures S1–S12). We can clearly see that by the composition ratios of Cs and Rb atoms (0%, 25%, 50%, 75% and 100%) can slightly affect the bandgaps of 2D HOIPs because the B-X bonds (B: Sn or Pb, X: Br or I) are changed (Figure 3a,b). As the concentration of Cs atom increases (Figure 3a), the bandgap of the Sn-based 2D-HOIP slightly decreases (from 1.19 to 1.12 eV and from 1.52 to 1.32 eV for SnI-2D-HOIPs and SnBr-2D-HOIPs, respectively). While for Pb-based systems (Figure 3a), the bandgap tends to slightly increase (from 2.01 to 2.08 eV and from 2.40 to 2.45 eV for PbI-2D-HOIPs and PbBr-2D-HOIPs, respectively). Different from the mixed Cs atom, as the concentration of Rb atom increases (Figure 3b), the bandgaps of all the four mixed 2D HOIPs system increase. We find that the smallest bandgap can be observed in SnI-2D-HOIP, and the largest one in PbBr-2D-HOIP at the same composition ratios of Cs and Rb atoms, which are the same as the pure 2D- and 3D-HOIPs [15]. We also find that the bandgap of 2D-HOIP mixed with Cs atom is smaller than that of the system mixed with Rb atom at the same composition ratios. So, SnI-2D-HOIPs by mixing Cs atom at the concentrations of 50%, 75% and 100% exhibit the smallest bandgap (1.12 eV, 1.13 eV, and 1.12 eV) (Figure 3a), respectively, while PbBr-2D-HOIPs by mixing Rb atom exhibits the largest bandgap at the concentration of 100% (2.59 eV) (Figure 3b).

The effects of metal atoms on the electronic properties are also investigated by replacing Sn by Ge and Pb with different composition ratios in the Sn-based 2D HOIPs. Different from Cs and Rb atom, the mixed Ge and Pb atoms play a key role on the electronic properties. Our calculations (Figure 3c) clearly show that the bandgaps of the mixed Sn-based 2D-HOIPs increase markedly as the concentrations of Ge and Pb atoms increase, except the SnI-2D-HOIPs and SnBr-2D-HOIPs at the concentration of Ge of 16.67%, where the bandgap is reduced from 1.19 to 1.07 eV and 1.52 to 1.49 eV, respectively. We also found that the SnI-2D-HOIPs with the mixed Ge/Pb atoms exhibit relatively small bandgaps compared to the SnBr-2D-HOIPs. Furthermore, the bandgap of Sn-based 2D-HOIPs by mixing Ge atom is smaller than that by mixing Pb atom at the same composition ratio. As a result, BA_2_MA_2_GeSn_2_I_10_ exhibits the smallest bandgap (1.07 eV), while BA_2_MA_2_Pb_2.5_Sn_0.5_Br_10_ is the largest one (2.02 eV) in these metal-atom mixed 2D-HOIPs.

To investigate the origin of the shift of band-edge states, we analyzed the partial densities of states that are calculated to find out the mechanism for the change of band edges (PDOSs) (Figures S1–S12). The CBBs of PbI-2D-HOIPs with the mixed Cs/Rb atoms (Figures S1f–j and S5f–j) mainly attribute to the Pb atoms’ p states. The VBTs are totally contributed by the p electrons from the I atoms at low concentration (0% and 25%) of the mixed Cs/Rb atoms, which is same as the pure PbI-2D-HOIPs [15]. As the concentration increases (50–100%), the VBTs is mainly contributed by the p electrons from I atoms and partially by the s electrons from the Pb atoms, while their CBBs keep unchanged. For PbBr-2D-HOIPs (Figures S2f–j and S6f–j), the mixed Cs/Rb atoms have no obvious effect on their CBBs and VBTs. The p electrons of the Br atoms dominate the VBTs with partial contribution from the s electrons of the Pb atoms. The p states of the Pb atoms dominate their CBBs. The p states of the Sn atoms contribute dominantly to the CBBs of For Sn-based 2D-HOIPs (Figures S3f–j, S4f–j, S7f–j and S8f–j), similar to the Pb-based 2D-HOIPs. In contrast, the p and s electrons from the halide atoms and Sn atoms, respectively, dominate their VBTs. In particular, as the concentration of Cs atom increases to 75% in SnI-2D-HOIPs, a defect band close to VBT is observable. It is found that the organic molecules, and the Cs and Rb atoms contribute negligibly to the band edge states in the considered systems.

Compared to the mixed Cs and Rb atoms, the mixed Ge and Pb atoms have a significant effect on the electronic properties of Sn-based 2D-HOIPs. The p electrons of the Sn and Ge atoms dominate the CBBs of SnI-2D-HOIPs (Figure S9f–j). As the concentrations of the Ge atom are 16.67% and 33.33%, the p electrons of the I atoms contribute dominantly to the VBTs. At a Ge concentration of 33.33%, a defect band is introduced into the bandgap. As the concentration increases, the p and s electrons of the I atom, and the (Sn and Ge) atoms, respectively, make contributions to the VBTs. Different from the Ge atoms, the mixed Pb atoms (Figure S11f–j) have no effect on the CBBs of SnI-based 2D HOIPs at the concentration of 16.67% and 33.33%. As the concentration increases (50%, 66.7%, and 83.3%), the p electrons of the Sn and Pb atoms dominate their CBBs. Their VBTs are contributed by the p electrons of I atoms and the s electrons of Sn and Pb atoms, respectively. Similarly, a defect band close to VBT is also observable at a concentration of 33.33%. The CBBs of SnBr-2D-HOIPs (Figure S10f–j) are dominated by the p electrons of the Sn and Ge atoms, similar to these of the SnI-2D-HOIPs. The p and s electrons from the I and (Sn and Ge) atoms, respectively, attribute to the VBTs. When the concentration is 83.33%, the p electrons of Ge atoms have a slight contribution to the VBTs edges states. The VBTs of the systems with the mixed Pb atom (Figure S12f–j) are mainly contributed by the s electrons of the Sn and Pb atoms and the p electrons of Br and Ge atoms. Their CBB is same as the SnI-based 2D HOIPs with the mixed Pb atoms.

### 3.3. Carrier Effective Mass

**Figure 4:**
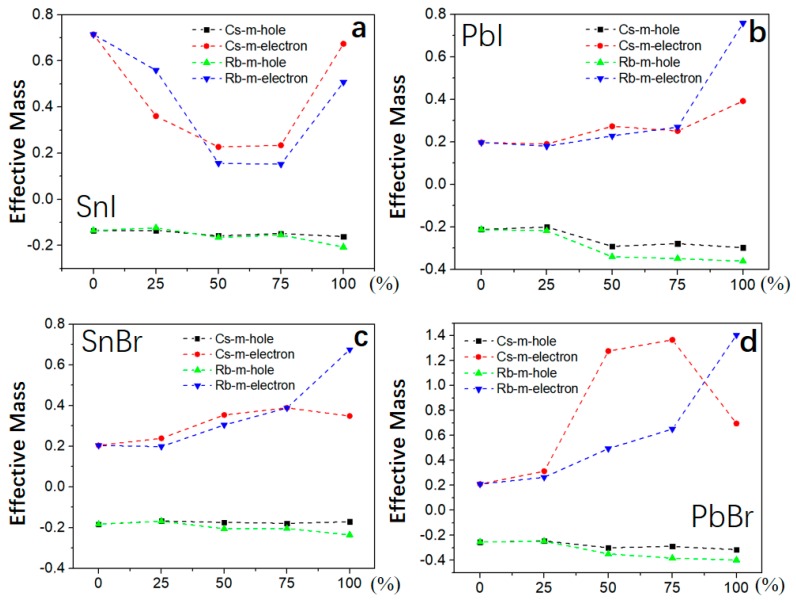
Carrier effective mass for 2D-HOIPs by mixing Cs/Rb atom at different concentrations (0%, 25%, 50%, 75% and 100%): (**a**) SnI-2D-HOIPs, (**b**) PbI-2D-HOIPs, (**c**) SnBr-2D-HOIPs, and (**d**) PbBr-2D-HOIPs.

**Figure 5:**
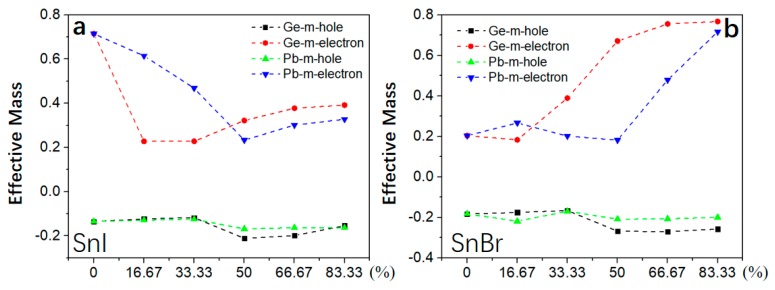
Carrier effective mass for 2D-HOIPs by mixing Ge/Pb atom at different concentrations (0%, 25%, 50%, 75% and 100%): (**a**) SnI-2D-HOIPs and (**b**) SnBr-2D-HOIPs.

As an important factor to characterize the efficiency of solar cells, carrier (electron and hole) effective masses (m_e_* and m_h_*m*) are calculated [39]. The m_h_* of 2D-HOIPs by mixing Cs/Rb atoms slightly decreases as the concentration of Cs/Rb atoms increases (Figure 4). The m_e_* exhibits an upward trend in PbI-2D-HOIPs, SnBr-2D-HOIPs and PbBr-2D-HOIPs (Figure 4b–d) as the concentration increases. For the SnI-based system (Figure 4a), m_e_* exhibits a downward trend as the concentration increases from 0 to 75%. In particular, m_e_* can be effectively reduced by 67.61% and 78.87% for the mixed Cs and Rb atom at a concentration of 50%, respectively (Figure 4a). Similarly, by mixing the Ge or Pb atoms into the SnI-based system (Figure 5a), m_e_* is also obviously reduced by 68.13% and 67.34% for Ge and Pb atom at the concentration of 16.67% and 50%, respectively. Differently, m_e_* of the SnBr-based system exhibits an upward trend as the concentration of Ge/Pb atoms increases (Figure 5b). As similar to the mixed Cs/Rb atoms systems, m_h_* slightly decreases with the concentration increased. We find that m_e_* is lower than the mixed Cs atom, while m_h_* shows no significant changes by mixing Rb atom into these four 2D HOIPs systems. In addition, the Sn-based system mixed with the Ge atom show a lower m_e_* than that mixed with the Pb atom at low concentration. As the concentration of Ge or Pb atom increases, m_e_* of Sn-based system mixed with the Ge atom is larger than that with the Pb atom. From the above analysis, it is clear that the mixed Cs, Rb, Ge, and Pb atoms have a significant effect on the electron effective mass of Sn-based 2D-HOIPs, especially for SnI-2D-HOIPs, which can effectively reduce its carrier effective mass by mixing the appropriate concentration. SnI-2D-HOIPs with the Rb atom at the concentration of 50% exhibits the lowest electron effective mass.

### 3.4. SnI-2D-HOIPs Mixed With Various Atoms (Cs, Rb, Ge, and Pb)

**Figure 6:**
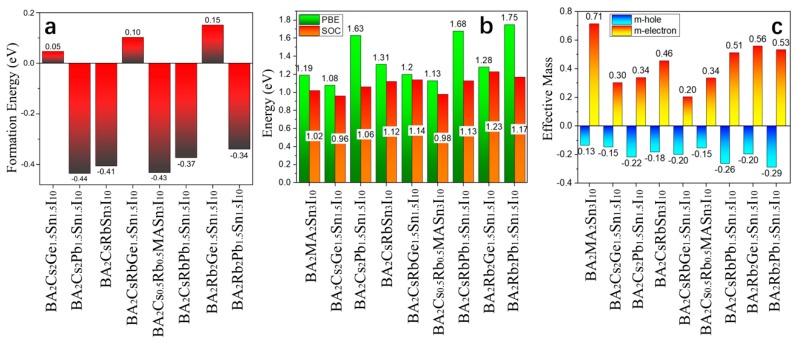
(**a**) Formation energies of the 2D SnI-based systems with various mixed atoms (Cs, Rb, Ge, and Pb). (**b**) Bandgaps of the 2D SnI-based systems with various mixed atoms (Cs, Rb, Ge, and Pb). (**c**) Carrier effective masses for the 2D SnI-based systems with various mixed atoms (Cs, Rb, Ge, and Pb).

Based on the discussion above, to further investigate the mixed 2D HOIPs, we also mixed the various atoms (Cs, Rb, Ge, and Pb) into the SnI-based system (Figures S13 and S14). It is found that the formation energies of the SnI-2D-HOIPs with Ge (BA_2_Cs_2_Ge_1.5_Sn_1.5_I_10_, BA_2_CsRbGe_1.5_Sn_1.5_I_10_, and BA_2_Rb_2_Ge_1.5_Sn_1.5_I_10_) are positive (Figure 6a). In these three systems, the Ge atom can more easily replace the Sn atom in BA_2_Cs_2_Sn_3_I_10_ than BA_2_CsRbSn_3_I_10_ and BA_2_Rb_2_Sn_3_I_10_ due to relatively low positive formation energy. While for the rest of the mixed 2D SnI-based HOIP systems, the formation energies are negative (Figure 6a). In other words, the Ge atom is more difficult to mix into the 2D-HOIPs than the Cs, Rb and Pb atom. The calculated bandgap (Figure 6b) of the SnI-2D-HOIPs with Ge is relatively small. The BA_2_Cs_2_Ge_1.5_Sn_1.5_I_10_ (Figure 6b,c) exhibits the smallest bandgap (1.08 eV) with small carrier effective mass (m_e_* = 0.30, m_h_* = −0.15) in the mixed compounds, which is almost equal to that of BA_2_MA_2_GeSn_2_I_10_ (1.07 eV) and smaller than those of the pure 2D SnI-based systems (1.19 eV). In particular, the bandgap of BA_2_Cs_0.5_Rb_0.5_MASn_3_I_10_ (Figure 6b,c) is the second-smallest (1.13 eV), which is also smaller than that of the pure system by 0.06 eV. Its effective mass is 0.3 and -0.15 for *m_e_* and m_h_*, respectively. BA_2_Rb_2_Pb_1.5_Sn_1.5_I_10_ exhibits the largest bandgap (1.75 eV) with large carrier effective masses (m_e_* = 0.53, m_h_* = −0.29). To confirm reliability of the results, the band structures with the spin–orbit coupling (SOC) are calculated (Figure 6b and Figure S15). We see that the bandgaps of the mixed SnI-2D-HOIPs with SOC show the same trend as those calculated from PBE-GGA method, except that BA_2_MA_2_GeSn_2_I_10_ (0.96 eV) and BA_2_Cs_0.5_Rb_0.5_MASn_3_I_10_ (0.98 eV) have smaller bandgaps than the pure one (1.02 eV).

### 3.5. Stability of the Mixed 2D-HOIPs

**Figure 7:**
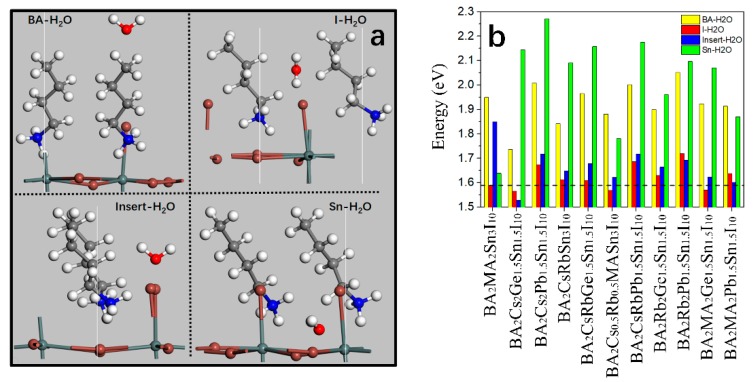
(**a**) Adsorption sites of water molecules. (**b**) Adsorption energies of water molecule for the various atoms (Cs, Rb, Ge, and Pb) mixed 2D SnI-based systems. The dashed line in (**b**) indicates the lowest H_2_O-adsorption energy on the pure 2D SnI-based system.

To investigate the stability of the mixed 2D-HOIPs, we calculated the adsorption energies of water molecules at all possible sites, including BA-H_2_O (near BA), Insert-H_2_O (between the two RNH_3_), I-H_2_O (near halide atom), and Sn-H_2_O (near metal atom) (Figure 7a). We focus on the SnI-2D-HOIPs mixed with different composition ratios of the Cs, Rb, Ge and Pb atoms. The energy for molecule adsorption (E_a_) can be calculated as follows [40]:

(2) $$E_{a}=E_{m/S}-E_{S}-E_{m}$$

where *E*_m/S_, *E*_S_, and *E*_m_ are the energies of the mixed 2D-HOIP with molecule, the 2D-HOIPs, and the molecule, respectively. If *E*_a_ is negative, the slab surface is an easy to adsorb molecule, while the surface is repulsive if the energy is positive. Similar to the pure 2D-HOIPs, our calculations (Figure 7b) show that all of the calculated *E*_a_ are positive in the considered structures (*E*_a_ in 3D-HOIPs are negative [55], confirming that the moisture is more difficult to penetrate into the mixed 2D-HOIPs than into 3D-HOIPs. In SnI-based systems with the various mixed atoms (Cs, Rb, Ge, and Pb), BA_2_CsRbPb_1.5_Sn_1.5_I_10_ and BA_2_Rb_2_Pb_1.5_Sn_1.5_I_10_ are the most stable to resist the water erosion (the lowest adsorption energy (1.69 eV) is larger than that of the pure system by 0.1 eV), while BA_2_Cs_2_Ge_1.5_Sn_1.5_I_10_ is easily eroded by the H_2_O molecule (the lowest adsorption energy (1.53 eV) is less than that of the pure system by 0.06 eV). We also find that the mixed Pb atoms can effectively enhance the stability of SnI-2D-HOIPs because of the E_a_. Therefore, we show that the SnI-2D-HOIPs by mixing the appropriate composition ratios of Cs, Rb, Ge, and Pb atoms can effectively improve the stability against H_2_O. Especially in BA_2_Cs_1.5_MA_0.5_Sn_3_I_10_, the lowest adsorption energy (1.9 eV) is much higher than other studied compounds, which is larger than the pure system by 0.31 eV, indicating its highest stability to resist effectively H_2_O.

## 4. Conclusions

In short, a DFT study on 2D BA_2_MA_2_B_3_X_10_ with various mixed atoms (Cs, Rb, Ge and Pb) and their physical properties and stabilities is presented. We show that SnI-2D-HOIPs by mixing different atoms has the lowest formation energy at the same composition ratio in four systems. Meanwhile, Cs, Rb, and Pb atoms can easily replace the corresponding molecule/atoms in 2D HOIPs under moderate conditions due to the negative substitutional energies, while Ge is difficult to replace Sn atom due to positive formation energy. We further show that the mixed atoms can effectively tune the electronic structures. For the mixed Cs and Rb atoms BA_2_CsMASn_3_I_10_, BA_2_Cs_1.5_MA_0.5_Sn_3_I_10_ and BA_2_Cs_2_Sn_3_I_10_ exhibit the smallest bandgaps (1.12 eV, 1.13 eV, and 1.12 eV). Furthermore, the mixed Cs, Rb, Ge, and Pb atoms have a significant effect on the electron effective mass of Sn-based 2D HOIPs system. Most especially, their carrier effective masses of SnI-based systems are significantly reduced by mixing the appropriate concentration of Cs/Rb/Ge/Pb. We show that mixing the appropriate composition ratios of Cs, Rb, Ge, and Pb atoms can effectively promote the performance of 2D HOIPs on the stability against water. For example, BA_2_Cs_1.5_MA_0.5_Sn_3_I_10_ with the bandgap of 1.13 eV and small carrier effective mass (m_e_* = 0.23, m_h_* = −0.14) exhibits high adsorption energy (>1.9 eV) in all studied compounds, where the lowest one is larger than that of the pure system by 0.31 eV. Compared with the 3D and pure-2D HOIPs, we demonstrate that the mixed 2D HOIPs with the appropriate composition ratios of Cs, Rb, Ge, and Pb atoms are more efficient in solar-to-power conversion because of reduced bandgaps and effective masses, and excellent stability on H_2_O. The findings confirm that BA_2_MA_2_B_3_X_10_ with the mixed atoms, especially BA_2_Cs_1.5_MA_0.5_Sn_3_I_10_, may have many practical applications in solar cells to replace 3D and pure 2D HOIPs.

## Supplementary Materials

The following are available online at https://www.mdpi.com/1420-3049/24/11/2144/s1, Tables S1–S4: Calculated lattice constants; Figures S1–S12: Calculated band structures and partial densities; Figures S13 and S15: Calculated band structures; Figure S14: Calculated partial densities.

## References

[1] Zhang W., Eperon G.E., Snaith H.J. (2016). Metal halide perovskites for energy applications. Nat. Energy.

[2] Saliba M., Matsui T., Domanski K., Seo J.-Y., Ummadisingu A., Zakeeruddin S.M., Correa-Baena J.-P., Tress W.R., Abate A., Hagfeldt A. (2016). Incorporation of rubidium cations into perovskite solar cells improves photovoltaic performance. Science.

[3] Cha H., Bae S., Lee M., Jeon H. (2016). Two-dimensional photonic crystal bandedge laser with hybrid perovskite thin film for optical gain. Appl. Phys. Lett..

[4] Byun J., Cho H., Wolf C., Jang M., Sadhanala A., Friend R.H., Yang H., Lee T.W. (2016). Efficient Visible Quasi-2D Perovskite Light-Emitting Diodes. Adv. Mater..

[5] Jeon N.J., Noh J.H., Yang W.S., Kim Y.C., Ryu S., Seo J., Seok S.I. (2015). Compositional engineering of perovskite materials for high-performance solar cells. Nature.

[6] Cao D.H., Stoumpos C.C., Farha O.K., Hupp J.T., Kanatzidis M.G. (2015). 2D Homologous Perovskites as Light-Absorbing Materials for Solar Cell Applications. J. Am. Chem. Soc..

[7] Niu W., Eiden A., Vijaya Prakash G., Baumberg J.J. (2014). Exfoliation of self-assembled 2D organic-inorganic perovskite semiconductors. Appl. Phys. Lett..

[8] Nagane S., Ogale S. (2016). CH_3_NH_3_Pb(BF_4_)_3_ and (C_4_H_9_NH_3_)_2_Pb(BF_4_)_4_ Family of 3D and 2D Perovskites without and with Iodide and Bromide Ions Substitution. J. Phys. Chem. Lett..

[9] Younts R., Duan H.S., Gautam B., Saparov B., Liu J., Mongin C., Castellano F.N., Mitzi D.B., Gundogdu K. (2017). Efficient Generation of Long-Lived Triplet Excitons in 2D Hybrid Perovskite. Adv. Mater..

[10] Tsai H., Nie W., Blancon J.-C., Stoumpos C.C., Asadpour R., Harutyunyan B., Neukirch A.J., Verduzco R., Crochet J.J., Tretiak S. (2016). High-efficiency two-dimensional Ruddlesden–Popper perovskite solar cells. Nature.

[11] Qiu W., Ray A., Jaysankar M., Merckx T., Bastos J.P., Cheyns D., Gehlhaar R., Poortmans J., Heremans P. (2017). An Interdiffusion Method for Highly Performing Cesium/Formamidinium Double Cation Perovskites. Adv. Funct. Mater..

[12] Yun S., Zhou X., Even J., Hagfeldt A. (2017). Theoretical Treatment of CH_3_NH_3_PbI_3_ Perovskite Solar Cells. Angew. Chem. Int. Ed..

[13] Hu H., Salim T., Chen B., Lam Y.M. (2016). Molecularly engineered organic-inorganic hybrid perovskite with multiple quantum well structure for multicolored light-emitting diodes. Sci. Rep..

[14] Dawson J.A., Naylor A.J., Eames C., Roberts M., Zhang W., Snaith H.J., Bruce P.G., Islam M.S. (2017). Mechanisms of lithium intercalation and conversion processes in organic–inorganic halide perovskites. ACS Energy Lett..

[15] Ma Z.-Q., Pan H., Wong P.K. (2016). A First-Principles Study on the Structural and Electronic Properties of Sn-Based Organic–Inorganic Halide Perovskites. J. Electron. Mater..

[16] Masi S., Rizzo A., Munir R., Listorti A., Giuri A., Esposito Corcione C., Treat N.D., Gigli G., Amassian A., Stingelin N. (2017). Organic Gelators as Growth Control Agents for Stable and Reproducible Hybrid Perovskite-Based Solar Cells. Adv. Energy Mater..

[17] Xiao M., Joglekar S., Zhang X., Jasensky J., Ma J., Cui Q., Guo L.J., Chen Z. (2017). Effect of Interfacial Molecular Orientation on Power Conversion Efficiency of Perovskite Solar Cells. J. Am. Chem. Soc..

[18] Yuan Z., Shu Y., Xin Y., Ma B. (2016). Highly luminescent nanoscale quasi-2D layered lead bromide perovskites with tunable emissions. Chem. Commun..

[19] Wang F., Geng W., Zhou Y., Fang H.H., Tong C.J., Loi M.A., Liu L.M., Zhao N. (2016). Phenylalkylamine Passivation of Organolead Halide Perovskites Enabling High-Efficiency and Air-Stable Photovoltaic Cells. Adv. Mater..

[20] Zhou C., Tian Y., Wang M., Rose A., Besara T., Doyle N.K., Yuan Z., Wang J.C., Clark R., Hu Y. (2017). Low-Dimensional Organic Tin Bromide Perovskites and Their Photoinduced Structural Transformation. Angew. Chem. Int. Ed..

[21] Kojima A., Teshima K., Shirai Y., Miyasaka T. (2009). Organometal Halide Perovskites as Visible-Light Sensitizers for Photovoltaic Cells. J. Am. Chem. Soc..

[22] Ji W., Jing P., Zhao J., Liu X., Wang A., Li H. (2013). Inverted CdSe/CdS/ZnS quantum dot light emitting devices with titanium dioxide as an electron-injection contact. Nanoscale.

[23] He Y., Galli G. (2017). Instability and Efficiency of Mixed Halide Perovskites CH_3_NH_3_AI_3–x_Cl_x_ (A = Pb and Sn): A First-Principles, Computational Study. Chem. Mater..

[24] Tong C.-J., Geng W., Tang Z.-K., Yam C.-Y., Fan X.-L., Liu J., Lau W.-M., Liu L.-M. (2015). Uncovering the Veil of the Degradation in Perovskite CH_3_NH_3_PbI_3_ upon Humidity Exposure: A First-Principles Study. J. Phys. Chem. Lett..

[25] Tang Z.K., Zhu Y.N., Xu Z.F., Liu L.M. (2017). Effect of water on the effective Goldschmidt tolerance factor and photoelectric conversion efficiency of organic-inorganic perovskite: Insights from first-principles calculations. Phys. Chem. Chem. Phys..

[26] Zhang L., Sit P.H.L. (2017). Ab initio study of the role of oxygen and excess electrons in the degradation of CH_3_NH_3_PbI_3_. J. Mater. Chem. A.

[27] Chen Y., Sun Y., Peng J., Zhang W., Su X., Zheng K., Pullerits T., Liang Z. (2017). Tailoring Organic Cation of 2D Air-Stable Organometal Halide Perovskites for Highly Efficient Planar Solar Cells. Adv. Energy Mater..

[28] Tang S., Deng Y., Zheng X., Bai Y., Fang Y., Dong Q., Wei H., Huang J. (2017). Composition Engineering in Doctor-Blading of Perovskite Solar Cells. Adv. Energy Mater..

[29] Wu Y., Xie F., Chen H., Yang X., Su H., Cai M., Zhou Z., Noda T., Han L. (2017). Thermally Stable MAPbI_3_ Perovskite Solar Cells with Efficiency of 19.19% and Area over 1 cm2 achieved by Additive Engineering. Adv. Mater..

[30] Guo Y., Li C., Li X., Niu Y., Hou S., Wang F. (2017). Effects of Rb Incorporation and Water Degradation on the Stability of the Cubic Formamidinium Lead Iodide Perovskite Surface: A First-Principles Study. J. Phys. Chem. C.

[31] Ferrara C., Patrini M., Pisanu A., Quadrelli P., Milanese C., Tealdi C., Malavasi L. (2017). Wide band-gap tuning in Sn-based hybrid perovskites through cation replacement: The FA_1__−x_MA_x_SnBr_3_ mixed system. J. Mater. Chem. A.

[32] Liu J., Wang G., Song Z., He X., Luo K., Ye Q., Liao C., Mei J. (2017). FAPb_1__−x_Sn_x_I_3_ mixed metal halide perovskites with improved light harvesting and stability for efficient planar heterojunction solar cells. J. Mater. Chem. A.

[33] Duong T., Wu Y., Shen H., Peng J., Fu X., Jacobs D., Wang E.-C., Kho T.C., Fong K.C., Stocks M. (2017). Rubidium Multication Perovskite with Optimized Bandgap for Perovskite-Silicon Tandem with over 26% Efficiency. Adv. Energy Mater..

[34] Yang Z., Rajagopal A., Chueh C.C., Jo S.B., Liu B., Zhao T., Jen A.K. (2016). Stable Low-Bandgap Pb-Sn Binary Perovskites for Tandem Solar Cells. Adv. Mater..

[35] Xu X., Chueh C.-C., Jing P., Yang Z., Shi X., Zhao T., Lin L.Y., Jen A.K.Y. (2017). High-Performance Near-IR Photodetector Using Low-Bandgap MA_0.5_FA_0.5_Pb_0.5_Sn_0.5_I_3_ Perovskite. Adv. Funct. Mater..

[36] Syzgantseva O.A., Saliba M., Gratzel M., Rothlisberger U. (2017). Stabilization of the Perovskite Phase of Formamidinium Lead Triiodide by Methylammonium, Cs, and/or Rb Doping. J. Phys. Chem. Lett..

[37] Zhang X., Liu H., Wang W., Zhang J., Xu B., Karen K.L., Zheng Y., Liu S., Chen S., Wang K. (2017). Hybrid Perovskite Light-Emitting Diodes Based on Perovskite Nanocrystals with Organic-Inorganic Mixed Cations. Adv. Mater..

[38] Mosconi E., Umari P., De Angelis F. (2015). Electronic and optical properties of mixed Sn–Pb organohalide perovskites: A first principles investigation. J. Mater. Chem. A.

[39] Ju M.G., Dai J., Ma L., Zeng X.C. (2017). Lead-Free Mixed Tin and Germanium Perovskites for Photovoltaic Application. J. Am. Chem. Soc..

[40] Ma Z.-Q., Shao Y., Wong P.K., Shi X., Pan H. (2018). Structural and Electronic Properties of Two-Dimensional Organic–inorganic Halide Perovskites and their Stability against Moisture. J. Phys. Chem. C.

[41] Zhu X., Xu Z., Zuo S., Feng J., Wang Z., Zhang X., Zhao K., Zhang J., Liu H., Priya S. (2018). Vapor-fumigation for record efficiency two-dimensional perovskite solar cells with superior stability. Energy Environ. Sci..

[42] Kim J.-H., Yang H. (2016). High-efficiency Cu–In–S quantum-dot-light-emitting device exceeding 7%. Chem. Mater..

[43] Hohenberg P., Kohn W. (1964). Inhomogeneous electron gas. Phys. Rev..

[44] Zamzam N., van Thor J.J. (2019). Excited State Frequencies of Chlorophyll f and Chlorophyll a and Evaluation of Displacement through Franck-Condon Progression Calculations. Molecules.

[45] Bennett J.W., Raglione M.E., Oburn S.M., MacGillivray L.R., Arnold M.A., Mason S.E. (2019). DFT Computed Dielectric Response and THz Spectra of Organic Co-Crystals and Their Constituent Components. Molecules.

[46] Tada K., Maruyama T., Koga H., Okumura M., Tanaka S. (2019). Extent of Spin Contamination Errors in DFT/Plane-wave Calculation of Surfaces: A Case of Au Atom Aggregation on a MgO Surface. Molecules.

[47] Blöchl P.E. (1994). Projector augmented-wave method. Phys. Rev. B.

[48] Perdew J.P., Burke K., Ernzerhof M. (1996). Generalized gradient approximation made simple. Phys. Rev. Lett..

[49] Kresse G., Joubert D. (1999). From ultrasoft pseudopotentials to the projector augmented-wave method. Phys. Rev. B.

[50] Kresse G., Furthmüller J. (1996). Efficient iterative schemes for ab initio total-energy calculations using a plane-wave basis set. Phys. Rev. B.

[51] Monkhorst H.J., Pack J.D. (1976). Special points for Brillouin-zone integrations. Phys. Rev. B.

[52] Zhang K., Zhang P., Wang Z.-R., Zhu X.-L., Lu Y.-B., Guan C.-B., Li Y. (2018). DFT simulations of the vibrational spectrum and hydrogen bonds of ice XIV. Molecules.

[53] Dion M., Rydberg H., Schröder E., Langreth D., Lundqvist B. (2005). Erratum: Van der Waals density functional for general geometries [Phys. Rev. Lett. 92, 246401 (2004)]. Phys. Rev. Lett..

[54] Mitzi D.B. (2001). Templating and structural engineering in organic-inorganic perovskites. J. Chem. Soc. Dalton Trans..

[55] Hao W., Chen X., Li S. (2016). Synergistic Effects of Water and Oxygen Molecule Co-adsorption on (001) Surfaces of Tetragonal CH_3_NH_3_PbI_3_: A First-Principles Study. J. Phys. Chem. C.

