# Supplementary material for: Mixed Two-Dimensional Organic-Inorganic Halide Perovskites for Highly Efficient and Stable Photovoltaic Application
Source: Molecules. 2019 Jun 6;24(11):2144. doi: 10.3390/molecules24112144 (PMC6600146; doi:10.3390/molecules24112144)
Supplement: Supplementary file 1 [file molecules-24-02144-s001.pdf]

molecules

Supporting Information

# Mixed Two-Dimensional Organic-Inorganic Halide Perovskites for Highly Efficient and Stable Photovoltaic Application

**Jia-Yi Dong [1], Zi-Qian Ma [2], Ye Yang [1], Shuang-Peng Wang [1,3,*] and Hui Pan [1,3,*]**

[1] Joint Key Laboratory of the Ministry of Education, Institute of Applied Physics and Materials Engineering, University of Macau, Macao SAR, 999078, China; yb67446@connect.um.edu.mo (J.-Y.D); yeyang@um.edu.mo (Y.Y.)

[2] School of Mechanical Engineering, Zhuhai College of Jilin University, Zhuhai, 519000, China; Yb47433@connect.umac.mo (Z.-Q.M.)

[3] Department of Physics and Chemistry, Faculty of Science and Technology, University of Macau, Macao SAR, 999078, China

* Correspondence: spwang@um.edu.mo (S.-P.W.); huipan@um.edu.mo (H.P.); Tel.: +85-38-822-4427 (H.P.); Fax: +85-38-822-2425 (H.P.)

**Table S1.** Calculated lattice constants (Å) of $BA_2MA_2B_3X_{10}$ (B = $Pb^{2+}$ or $Sn^{2+}$; X = $Br^-$ or $I^-$) with the mixed Cs atom.

| Rb atom | SnI-based | | | PbI-based | | | SnBr-based | | | PbBr-based | | |
|---|---|---|---|---|---|---|---|---|---|---|---|---|
| | a | b | c | a | b | c | a | b | c | a | b | c |
| 0% | 8.83 | 8.83 | 26.57 | 8.92 | 8.92 | 26.69 | 8.34 | 8.34 | 25.55 | 8.42 | 8.42 | 25.65 |
| 25% | 8.79 | 8.79 | 26.5 | 8.91 | 8.91 | 26.63 | 8.33 | 8.33 | 25.28 | 8.4 | 8.4 | 25.61 |
| 50% | 8.8 | 8.8 | 26.34 | 8.82 | 8.82 | 26.76 | 8.31 | 8.31 | 25.17 | 8.3 | 8.3 | 25.59 |
| 75% | 8.77 | 8.77 | 26.3 | 8.76 | 8.76 | 26.64 | 8.24 | 8.24 | 25.18 | 8.26 | 8.26 | 25.58 |
| 100% | 8.69 | 8.69 | 26.37 | 8.76 | 8.76 | 26.63 | 8.15 | 8.15 | 25.3 | 8.23 | 8.23 | 25.61 |

**Table S2.** Calculated lattice constants (Å) of $BA_2MA_2B_3X_{10}$ (B = $Pb^{2+}$ or $Sn^{2+}$; X = $Br^-$ or $I^-$) with the mixed Rb atom.

| Cs atom | SnI-based | | | PbI-based | | | SnBr-based | | | PbBr-based | | |
|---|---|---|---|---|---|---|---|---|---|---|---|---|
| | a | b | c | a | b | c | a | b | c | a | b | c |
| 0% | 8.83 | 8.83 | 26.57 | 8.92 | 8.92 | 26.69 | 8.34 | 8.34 | 25.55 | 8.42 | 8.42 | 25.65 |
| 25% | 8.84 | 8.84 | 26.49 | 8.85 | 8.85 | 26.75 | 8.33 | 8.33 | 25.44 | 8.4 | 8.4 | 25.65 |
| 50% | 8.83 | 8.83 | 26.44 | 8.85 | 8.85 | 26.77 | 8.33 | 8.33 | 25.27 | 8.33 | 8.33 | 25.7 |
| 75% | 8.82 | 8.82 | 26.4 | 8.83 | 8.83 | 26.97 | 8.32 | 8.32 | 25.24 | 8.33 | 8.33 | 25.7 |
| 100% | 8.8 | 8.8 | 26.48 | 8.8 | 8.8 | 26.85 | 8.28 | 8.28 | 25.15 | 8.28 | 8.28 | 25.75 |

**Table S3.** Calculated lattice constants (Å) of $BA_2MA_2Sn_3I_{10}$ with the mixed Ge atom.

| Ge atom | SnI-based | | | SnBr-based | | |
|---|---|---|---|---|---|---|
| | a | b | c | a | b | c |
| 0% | 8.83 | 8.83 | 26.57 | 8.34 | 8.34 | 25.55 |
| 16.67% | 8.83 | 8.83 | 26.42 | 8.33 | 8.33 | 25.37 |
| 33.33% | 8.81 | 8.81 | 26.35 | 8.33 | 8.33 | 25.22 |
| 50% | 8.75 | 8.75 | 26.25 | 8.29 | 8.29 | 25.18 |
| 66.67% | 8.72 | 8.72 | 26.17 | 8.25 | 8.25 | 25.25 |
| 83.33% | 8.71 | 8.71 | 26.04 | 8.24 | 8.24 | 25.23 |

**Table S4.** Calculated lattice constants (Å) of $BA_2MA_2Sn_3I_{10}$ with the mixed Pb atom.

| Pb atom | SnI-based | | | SnBr-based | | |
|---|---|---|---|---|---|---|
| | a | b | c | a | b | c |
| 0% | 8.83 | 8.83 | 26.57 | 8.34 | 8.34 | 25.55 |
| 16.67% | 8.86 | 8.86 | 26.54 | 8.31 | 8.31 | 25.51 |
| 33.33% | 8.88 | 8.88 | 26.57 | 8.38 | 8.38 | 25.53 |
| 50% | 8.89 | 8.89 | 26.59 | 8.38 | 8.38 | 25.53 |
| 66.67% | 8.89 | 8.89 | 26.65 | 8.41 | 8.41 | 25.53 |
| 83.33% | 8.91 | 8.91 | 26.77 | 8.4 | 8.4 | 25.63 |

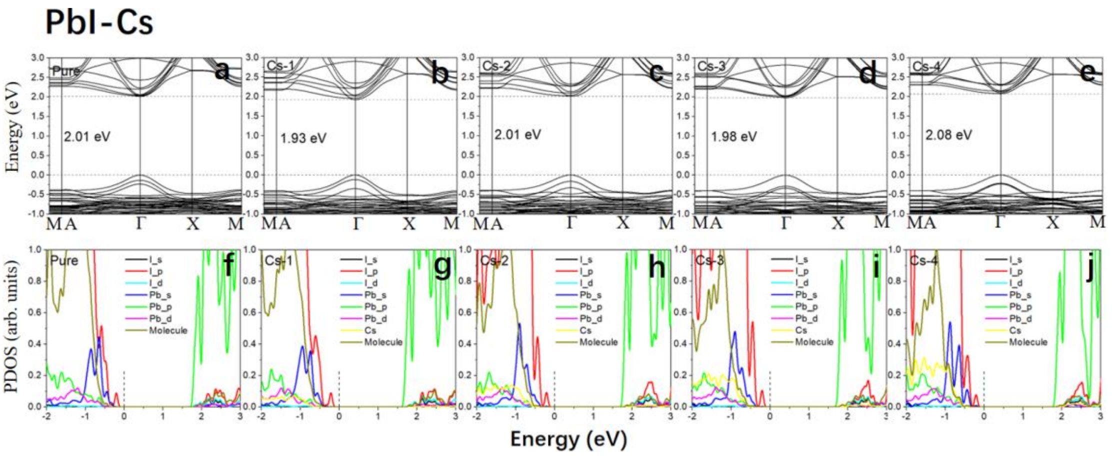

**Figure S1.** Calculated band structures of PbI-based 2D HOIP by mixing Cs atom with different concentrations: (a) pure (0%), (b) Cs-1 (25%), (c) Cs-2 (50%), and (d) Cs-3 (75%), (e) Cs-4 (100%). Calculated partial densities of states (PDOSs) of PbI-based 2D HOIP by mixing Cs atom with different concentrations: (f) pure, (g) Cs-1, (h) Cs-2, (i) Cs-3, and (j) Cs-4.

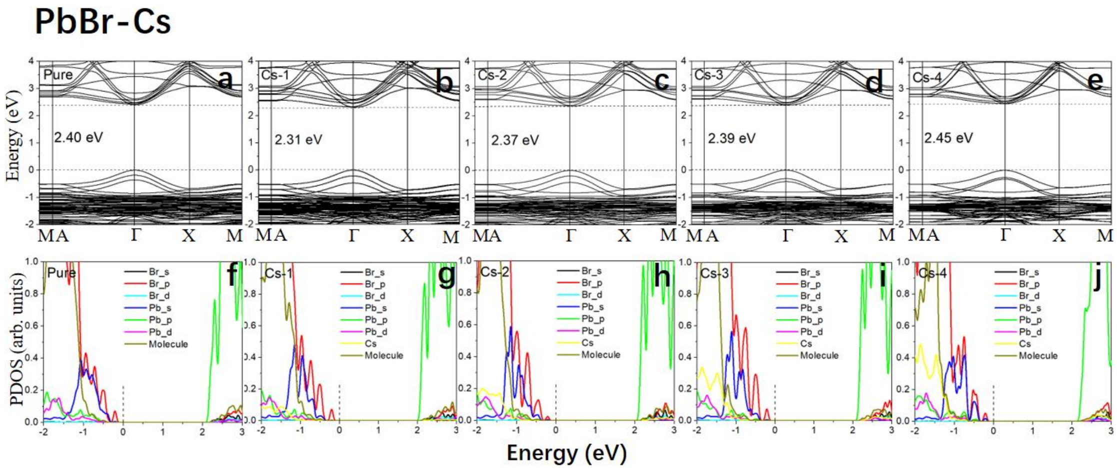


**Figure S2.** Calculated band structures of PbBr-based 2D HOIP by mixing Cs atom with different concentrations: (a) pure, (b) Cs-1, (c) Cs-2, (d) Cs-3, and (e) Cs-4. Calculated partial densities of states (PDOSs) of PbBr-based 2D HOIP by mixing Cs atom with different concentrations: (f) pure, (g) Cs-1, (h) Cs-2, (i) Cs-3, and (j) Cs-4.

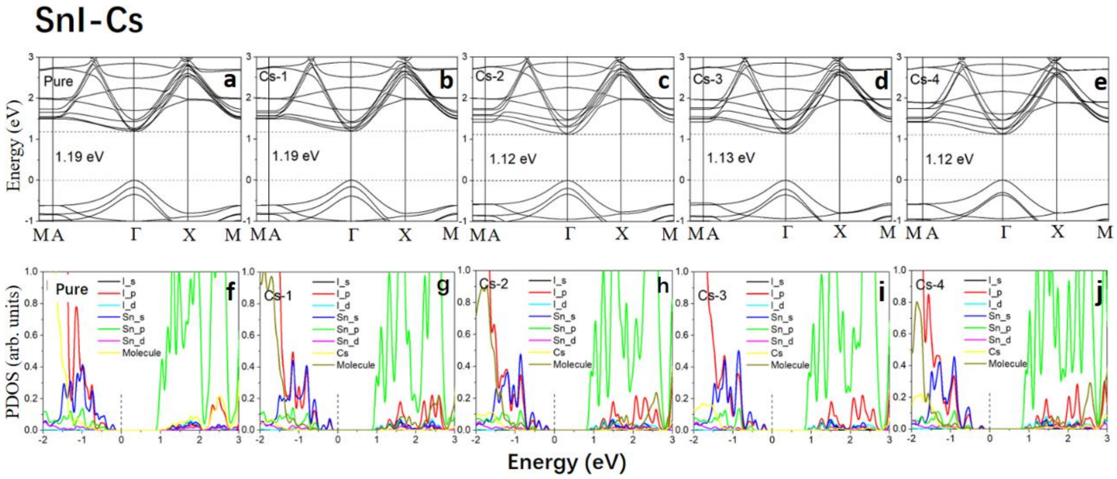


**Figure S3.** Calculated band structures of SnI-based 2D HOIP by mixing Cs atom with different concentrations: (a) pure, (b) Cs-1, (c) Cs-2, (d) Cs-3, and (e) Cs-4. Calculated partial densities of states (PDOSs) of SnI-based 2D HOIP by mixing Cs atom with different concentrations: (f) pure, (g) Cs-1, (h) Cs-2, (i) Cs-3, and (j) Cs-4.

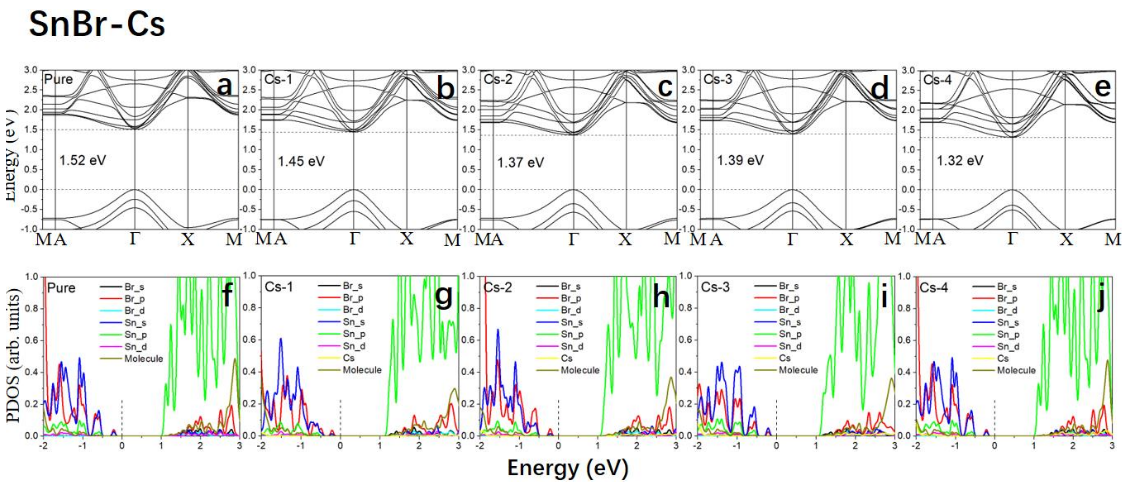


**Figure S4.** Calculated band structures of SnBr-based 2D HOIP by mixing Cs atom with different concentrations: (a) pure, (b) Cs-1, (c) Cs-2, (d) Cs-3, and (e) Cs-4. Calculated partial densities of states (PDOSs) of SnBr-based 2D HOIP by mixing Cs atom with different concentrations: (f) pure, (g) Cs-1, (h) Cs-2, (i) Cs-3, and (j) Cs-4.

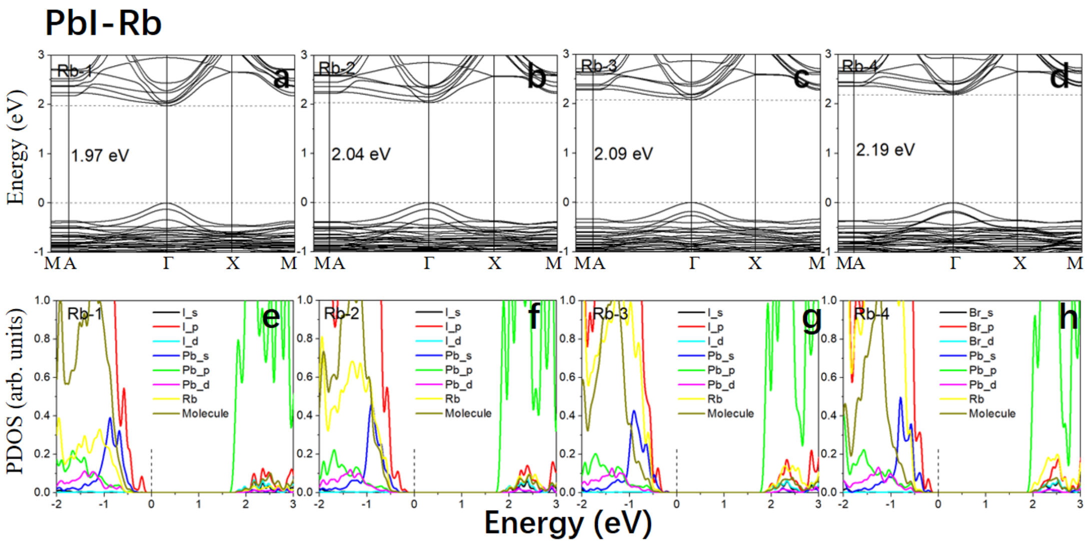


**Figure S5.** Calculated band structures of PbI-based 2D HOIP by mixing Rb atom with different concentrations: (a) Rb-1 (25%), (b) Rb-2 (50%), (c) Rb-3 (75%), and (d) Rb-4 (100%). Calculated partial densities of states (PDOSs) of PbI-based 2D HOIP by mixing Rb atom with different concentrations: (e) Rb-1, (f) Rb-2, (g) Rb-3, and (h) Rb-4.

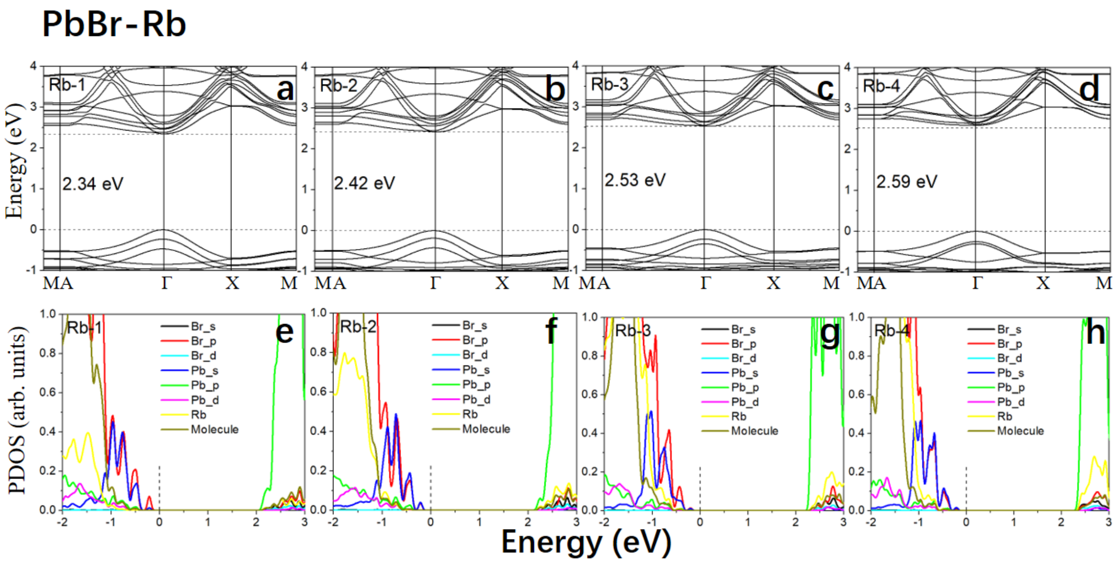


**Figure S6.** Calculated band structures of PbBr-based 2D HOIP by mixing Rb atom with different concentrations: (a) Rb-1, (b) Rb-2, (c) Rb-3, and (d) Rb-4. Calculated partial densities of states (PDOSs) of PbBr-based 2D HOIP by mixing Rb atom with different concentrations: (e) Rb-1, (f) Rb-2, (g) Rb-3, and (h) Rb-4.

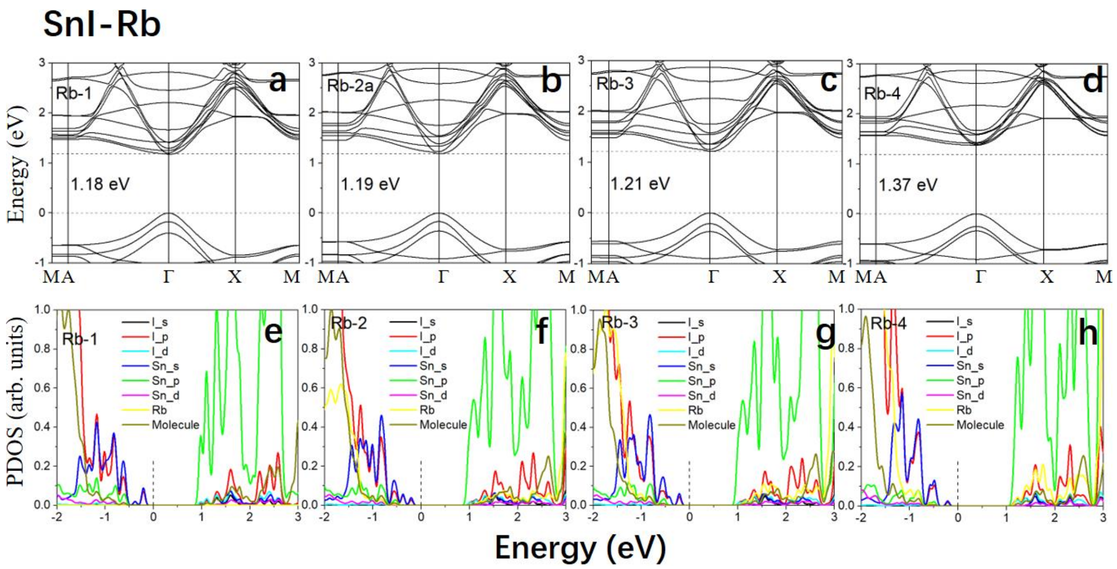


**Figure S7.** Calculated band structures of SnI-based 2D HOIP by mixing Rb atom with different concentrations: (a) pure, (b) Rb-1, (c) Rb-2, (d) Rb-3. Calculated partial densities of states (PDOSs) of SnI-based 2D HOIP by mixing Rb atom with different concentrations: (e) pure, (f) Rb-1, (g) Rb-2, and (h) Rb-3.

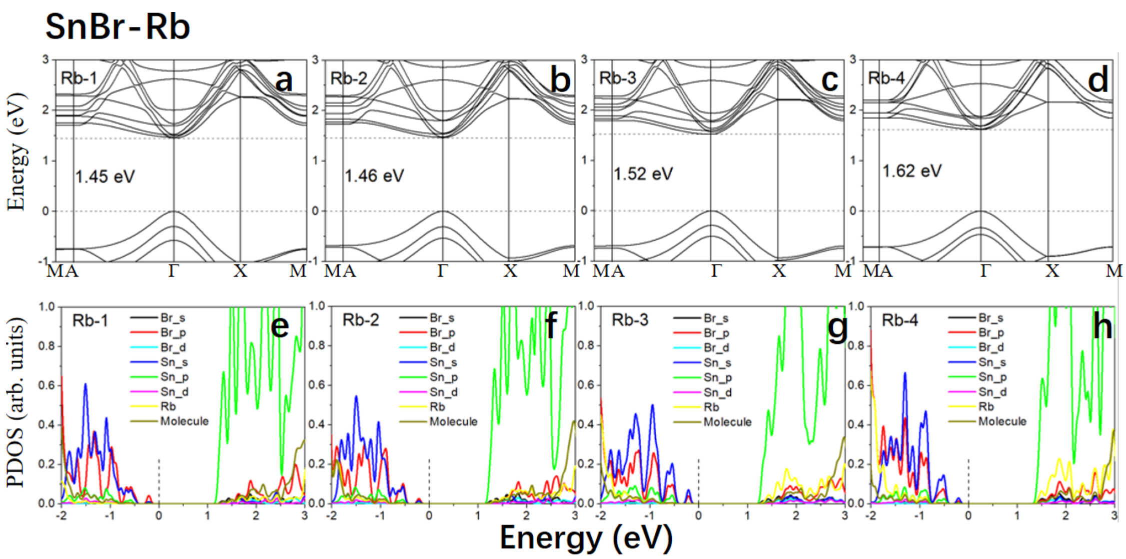


**Figure S8.** Calculated band structures of SnBr-based 2D HOIP by mixing Rb atom with different concentrations: (a) Rb-1, (b) Rb-2, (c) Rb-3, (d) Rb-4. Calculated partial densities of states (PDOSs) of SnBr-based 2D HOIP by mixing Rb atom with different concentrations: (e) Rb-1, (f) Rb-2, (g) Rb-3, and (h) Rb-4.

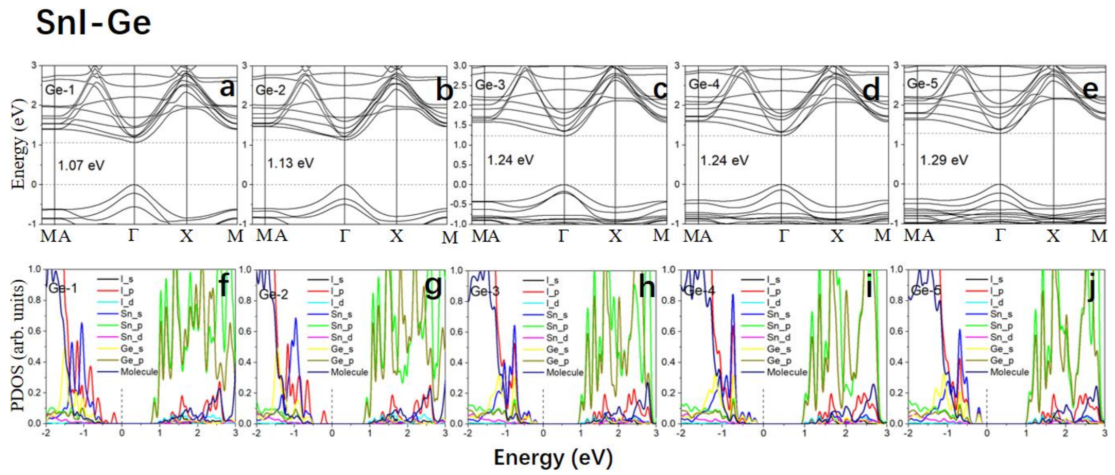


**Figure S9.** Calculated band structures of SnI-based 2D HOIP by mixing Ge atom with different concentrations: (a) Ge-1 (16.67%), (b) Ge-2 (33.33%), (c) Ge-3 (50%), (d) Ge-4 (66.67%), (e) Ge-5 (83.33%). Calculated partial densities of states (PDOSs) of SnI-based 2D HOIP by mixing Ge atom with different concentrations: (f) Ge-1, (g) Ge-2, (h) Ge-3, (i) Ge-4, and (j) Ge-5.

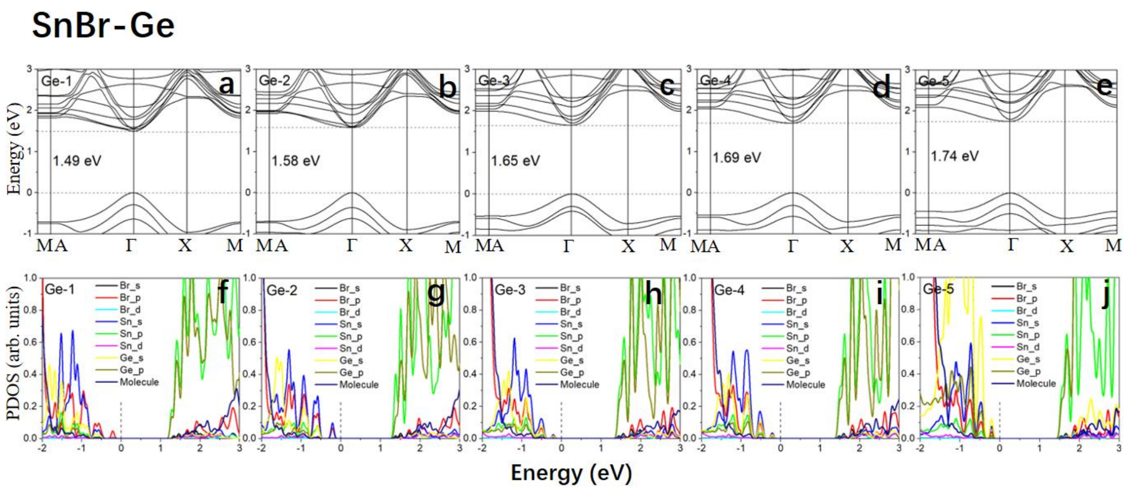

**Figure S10.** Calculated band structures of SnBr-based 2D HOIP by mixing Ge atom with different concentrations: (a) Ge-1, (b) Ge-2, (c) Ge-3, (d) Ge-4, (e) Ge-5. Calculated partial densities of states (PDOSs) of SnBr-based 2D HOIP by mixing Ge atom with different concentrations: (f) Ge-1, (g) Ge-2, (h) Ge-3, (i) Ge-4, and (j) Ge-5.

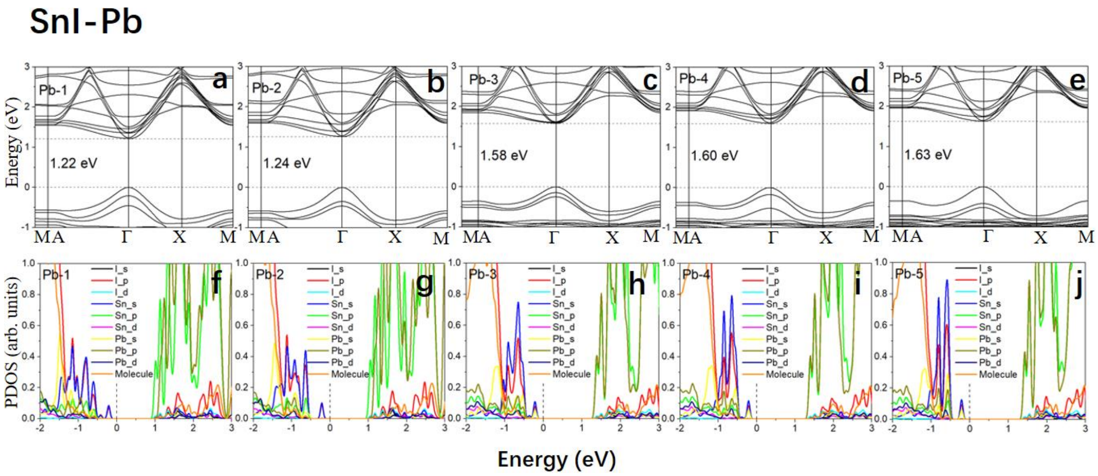


**Figure S11.** Calculated band structures of SnI-based 2D HOIP by mixing Pb atom with different concentrations: (a) Pb-1 (16.67%), (b) Pb-2 (33.33%), (c) Pb-3 (50%), (d) Pb-4 (66.67%), and (e) Pb-5 (83.33%). Calculated partial densities of states (PDOSs) of SnI-based 2D HOIP by mixing Pb atom with different concentrations: (f) Pb-1, (g) Pb-2, (h) Pb-3, (i) Pb-4, and (j) Pb-5.

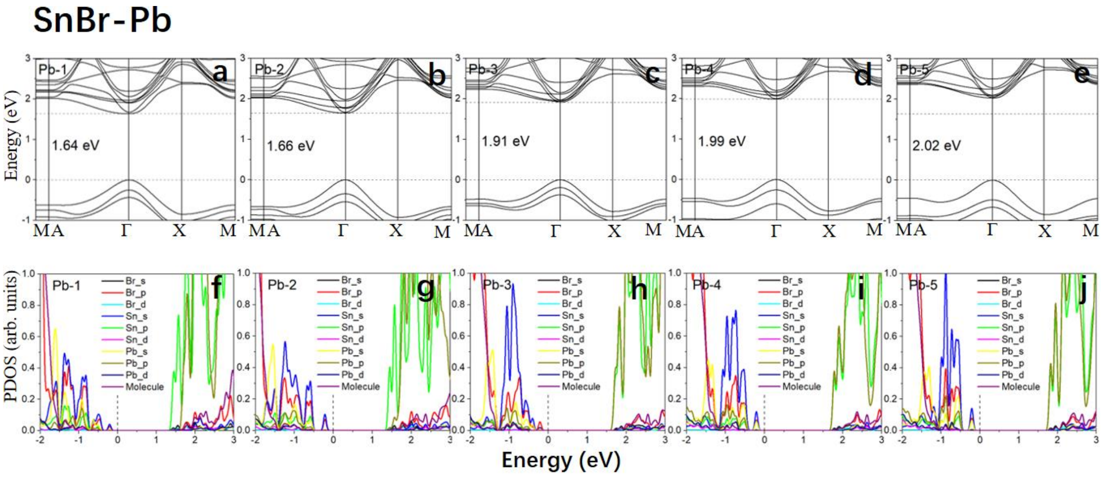


**Figure S12.** Calculated band structures of SnBr-based 2D HOIP by mixing Pb atom with different concentrations: (a) Pb-1, (b) Pb-2, (c) Pb-3, (d) Pb-4, (e) Pb-5. Calculated partial densities of states (PDOSs) of SnBr-based 2D HOIP by mixing Pb atom with different concentrations: (f) Pb-1, (g) Pb-2, (h) Pb-3, (i) Pb-4, and (j) Pb-5.

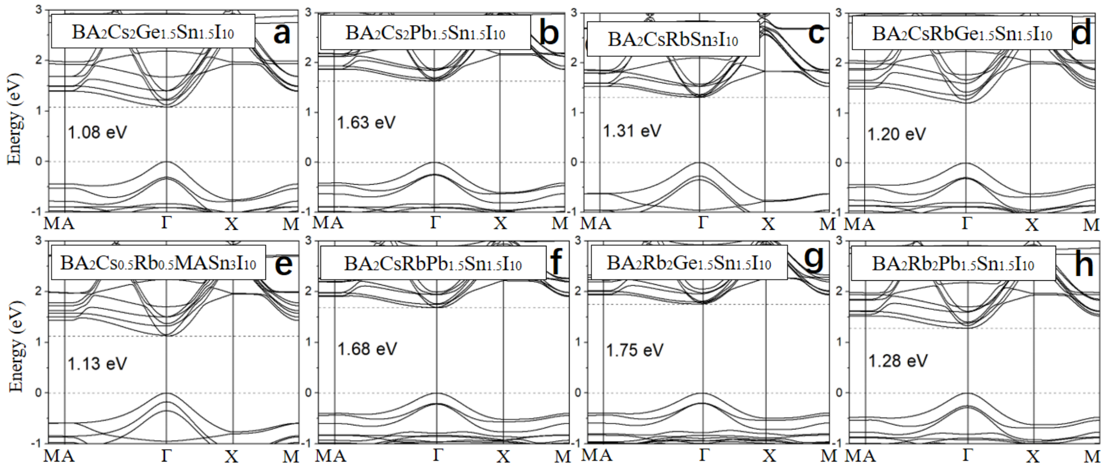


**Figure S13.** Calculated band structures of the various atoms (Cs, Rb, Ge, and Pb) mixed 2D SnI-based system: (a) $BA_2Cs_2Ge_{1.5}Sn_{1.5}I_{10}$, (b) $BA_2Cs_2Pb_{1.5}Sn_{1.5}I_{10}$, (c) $BA_2CsRbSn_3I_{10}$, (d) $BA_2CsRbGe_{1.5}Sn_{1.5}I_{10}$, (e) $BA_2Cs_{0.5}Rb_{0.5}MASn_3I_{10}$, (f) $BA_2CsRbPb_{1.5}Sn_{1.5}I_{10}$, (g) $BA_2Rb_2Ge_{1.5}Sn_{1.5}I_{10}$, and (h) $BA_2Rb_2Pb_{1.5}Sn_{1.5}I_{10}$.

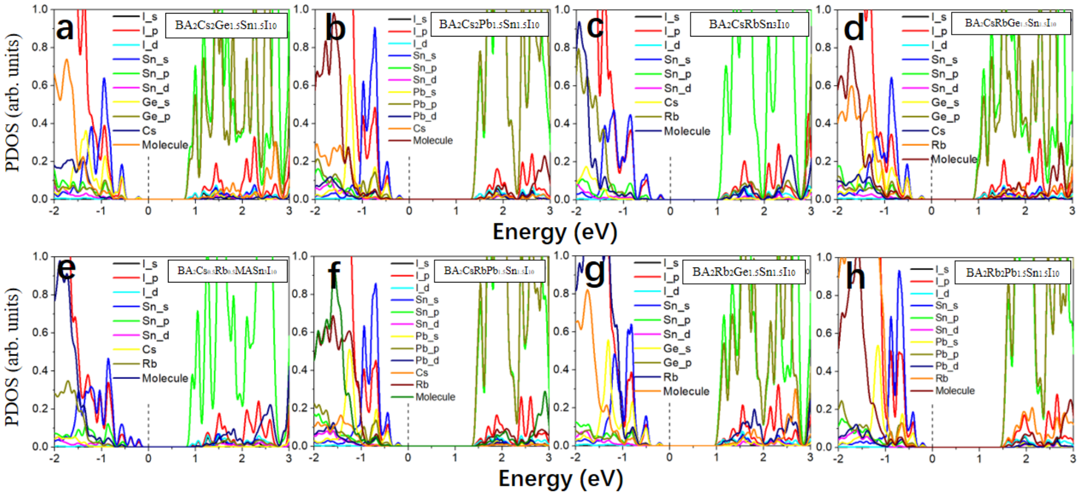


**Figure S14.** Calculated partial densities of states (PDOSs) of the various atoms (Cs, Rb, Ge, and Pb) mixed 2D SnI-based system: (a) $BA_2Cs_2Ge_{1.5}Sn_{1.5}I_{10}$, (b) $BA_2Cs_2Pb_{1.5}Sn_{1.5}I_{10}$, (c) $BA_2CsRbSn_3I_{10}$, (d) $BA_2CsRbGe_{1.5}Sn_{1.5}I_{10}$, (e) $BA_2Cs_{0.5}Rb_{0.5}MASn_3I_{10}$, (f) $BA_2CsRbPb_{1.5}Sn_{1.5}I_{10}$, (g) $BA_2Rb_2Ge_{1.5}Sn_{1.5}I_{10}$, and (h) $BA_2Rb_2Pb_{1.5}Sn_{1.5}I_{10}$.

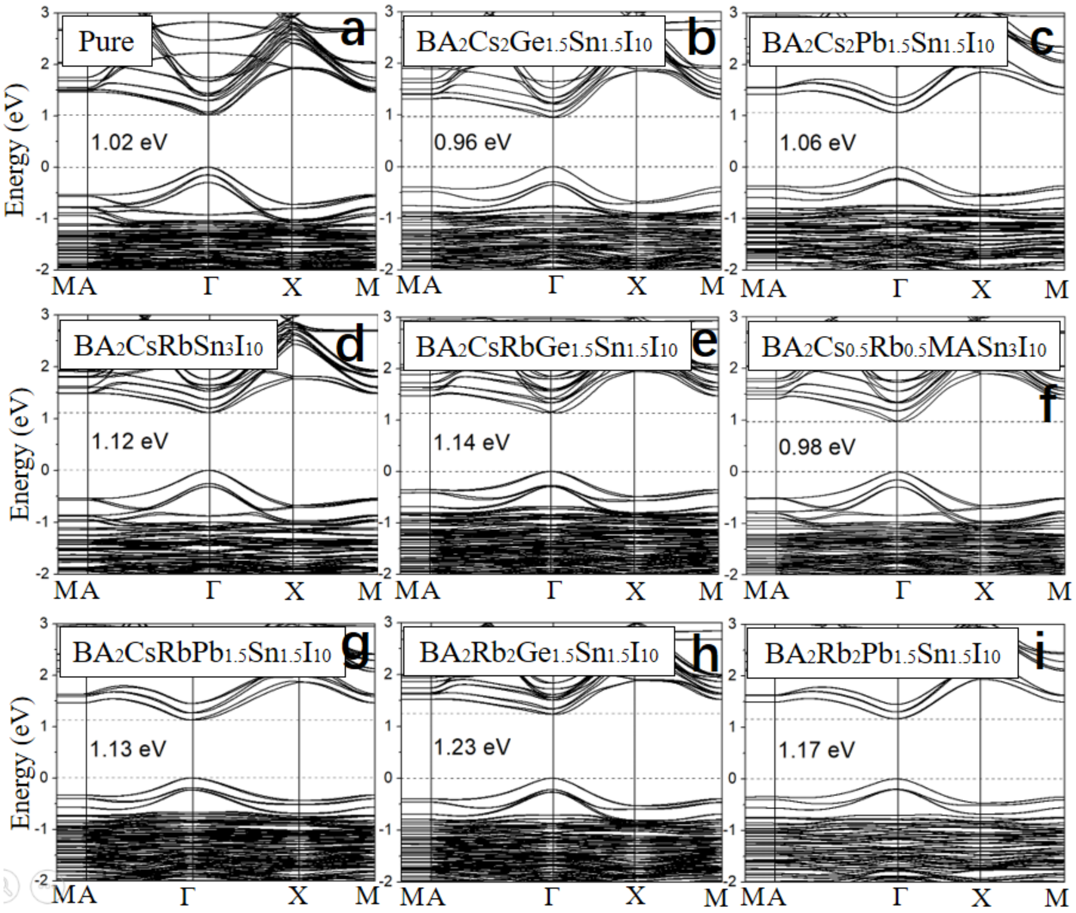


**Figure S15.** Calculated band structures with SOC effect for the various atoms (Cs, Rb, Ge, and Pb) mixed 2D SnI-based system: (a)Pure, (b) $BA_2Cs_2Ge_{1.5}Sn_{1.5}I_{10}$, (c) $BA_2Cs_2Pb_{1.5}Sn_{1.5}I_{10}$, (d) $BA_2CsRbSn_3I_{10}$, (e) $BA_2CsRbGe_{1.5}Sn_{1.5}I_{10}$, (f) $BA_2Cs_{0.5}Rb_{0.5}MASn_3I_{10}$, (g) $BA_2CsRbPb_{1.5}Sn_{1.5}I_{10}$, (h) $BA_2Rb_2Ge_{1.5}Sn_{1.5}I_{10}$, and (i) $BA_2Rb_2Pb_{1.5}Sn_{1.5}I_{10}$.
